# Interdisciplinary approach allows minimally invasive, nerve-sparing removal of retroperitoneal peripheral nerve sheath tumors

**DOI:** 10.1007/s00423-019-01851-5

**Published:** 2020-01-11

**Authors:** Mohammed Mehdi Hajiabadi, Benito Campos, Oliver Sedlaczek, Elias Khajeh, Mohammadsadegh Nikdad, Andreas von Deimling, Arianeb Mehrabi, Andreas Unterberg, Rezvan Ahmadi

**Affiliations:** 1 Department of Neurosurgery, University Hospital Heidelberg, INF 400, 69120 Heidelberg, Germany; 2 Department of Radiology, University Hospital Heidelberg, Heidelberg, Germany; 3 Department of General, Visceral, and Transplantation Surgery, University Hospital Heidelberg, Heidelberg, Germany; 4 Department of Neuropathology, University Hospital Heidelberg, Heidelberg, Germany; 5 Clinical Cooperation Unit Neuropathology and German Cancer Research Center (DKFZ), Heidelberg, Germany

**Keywords:** Retroperitoneal, Nerve sheath tumor, Interdisciplinary, Nerve-sparing

## Abstract

**Purpose:**

En bloc resection of retroperitoneal peripheral nerve sheath tumors (PNST) is advocated by a variety of surgical disciplines. Yet, microsurgical, nerve-sparing tumor resection might be better suited to improve symptoms and maintain neurological function, especially in cases where patients present with preoperative neurological deficits. However, neurosurgeons, versed in nerve-sparing techniques to remove PNST, are generally unfamiliar with the visceral approaches to retroperitoneal PNST.

**Methods:**

We retrospectively evaluate a series of 16 patients suffering from retroperitoneal PNST. Patients were treated by a unique interdisciplinary approach, combining the visceral surgeon’s skills to navigate the complex anatomy of the retroperitoneal space and the neurosurgeon’s familiarity with microsurgical, nerve-sparing tumor removal. Specifically, we assess whether our interdisciplinary approach is suited to improve preoperative symptoms and maintain neurological function and study whether oncological outcome, surgical morbidity, and operative times are comparable to those reported for “classical” retroperitoneal PNST resection. In addition, we study two cases of suspected PNST that were diagnosed as malignant peripheral nerve sheath tumors (MPNST) after surgery.

**Results:**

Total macroscopic tumor resection was achieved in 14/16 PNST patients. Mean intraoperative blood loss was 680.6 ml (95% CI, 194.3–1167.0 ml) and mean operative time was 162.5 min (95% CI, 121.6–203.4 min). We did not record any major postoperative surgical or neurological complications. A total of 8/11 patients with preoperative pain symptoms reported long-lasting improvement of their symptoms. In terms of oncological outcome, all patients that had been subjected to total tumor removal and for whom follow-up was available, were tumor-free after a mean follow-up of 761.9 days (95% CI, 97.6–1426.0 days). One of the two MPNST patients, who presented with tumor progress 15 months after initial surgery, was subjected to radical re-resection.

**Conclusions:**

Interdisciplinary, nerve-sparing removal of retroperitoneal PNST is well suited to improve preoperative symptoms and maintain neurological function, while achieving an oncological outcome and a surgical morbidity similar to previously reported results for radical retroperitoneal PNST resection. Radical re-resection was feasible in a patient with post hoc MPNST diagnosis.

## Introduction

Neurosurgeons have developed and refined microsurgical, nerve-sparing techniques to remove benign peripheral nerve sheath tumors (PNST) [1]. Typically, PNST surgery involves the following steps: (1) identification of the tumor pseudocapsule consisting of the epineurium and viable nerve fascicles which are stretched and thinned out around the tumor; (2) incision of the pseudocapsule, avoiding viable nerve fascicles in the pseudocapsule; and (3) extirpation of the tumor after dissection of the plane between the pseudocapsule and the true tumor capsule.

These techniques have enabled neurosurgeons to perform gross total tumor removal of PNST in almost every localization while improving preoperative symptoms, preserving neurological function, and obtaining excellent oncological results [2].

Retroperitoneal PNST, originating from lumbosacral nerve roots or the lumbar plexus, are a notable exception. Given that PNST are just one of the many potential tumor varieties in the retroperitoneal space [3], retroperitoneal PNST are typically treated by surgeons from other disciplines such as visceral surgery and oncological surgery.

There are two main strategies in dealing with histology-proven PNST, specifically, en bloc resection with or without margin resection and radiological monitoring. A recent retrospective multicenter study studied growth dynamics of 248 retroperitoneal PNST, which were initially subjected to radiological monitoring [4]. The average annual growth rate was 10.5%, prompting the authors to recommend early surgery in cases of symptomatic tumor, diagnostic uncertainty, and existing evidence of rapid expansion or patient preference after adequate counseling.

Complete excision is the therapy of choice but considerable controversy exists over timing of surgery and negative soft tissue margins [4, 5]. Aggressive en bloc resection to avoid local tumor recurrence and/or malignant transformation is recommended by national guidelines, such as the German guidelines for malignant soft tissue tumors. In addition, guidelines [6] recommend a one-specimen en bloc resection as the surgery of choice for histology-proven retroperitoneal malignant peripheral nerve sheath tumors (MPNST). Ideally, this includes resection of adherent structures, even if not evidently infiltrated. Preservation of specific organs/neurovascular structures should be considered on an individualized basis. En bloc resection of the tumor, however, inevitably leads to loss of function of viable nerve fascicles in the tumor pseudocapsule. Even though some authors advocate a less radical retroperitoneal PNST resection to limit neurological morbidity [5], this approach typically still involves resection of nerve fascicles trapped in the tumor pseudocapsule and can be associated with severe impairment of neurological function.

There is a void of information regarding neurological outcome after retroperitoneal PNST resection. It is likely, however, that neurological morbidity after radical retroperitoneal PNST resection is substantially higher than neurological morbidity after neurosurgical, nerve-sparing PNST removal.

In our department, retroperitoneal PNST are routinely treated by an interdisciplinary team consisting of a visceral surgeon and a neurosurgeon, i.e., combining the general surgeon’s skills to navigate the complex anatomy of the retroperitoneal space and the neurosurgeon’s familiarity with microsurgical, nerve-sparing tumor resections. We hypothesize that such an interdisciplinary approach is best suited to improve preoperative symptoms and maintain neurological function [7] and offers the possibility to move from a microsurgical, nerve-sparing resection to a more aggressive, oncological resection in rare cases were MPNST can be diagnosed with intraoperative histology. In addition, our study is motivated by three considerations: (1) Does retroperitoneal PNST resection achieve a similar oncological outcome compared with radical tumor resection?; (2) Is our minimally invasive tumor resection, associated with less surgical morbidity, e.g., less intraoperative blood loss? ; (3) Does our interdisciplinary approach prolong surgery compared with a monodisciplinary tumor resection?

Here, we conduct a retrospective evaluation of 18 patients suffering from retroperitoneal tumors, including 16 PNST and 2 MPNST, who were treated at our department between 2009 and 2018 by an interdisciplinary surgical team. Specifically, we evaluate neurological as well as oncological outcome, and study surgical morbidity and operative times.

## Material and methods

### Study design and surgical technique

During the study period (2009–2018), 18 patients suffering from retroperitoneal tumors, including 16 PNST and 2 MPNST, were treated by an interdisciplinary team consisting of a neurosurgeon and a general surgeon. For all 18 cases, we retrospectively compiled clinical parameters from medical records. Additionally, radiological reports, T1 and T2 weighted MRI imaging sets, and histopathological reports were extracted. Information was gathered according to the research proposals approved by the Institutional Review Board at the Medical Faculty Heidelberg.

All tumors were accessed via laparotomy or through a retroperitoneal approach in cases were the tumor extended through the foramen obturatum. While the neurosurgeon remained the same in all cases (RA), the general surgeon differed from surgery to surgery.

**Fig. 1:**
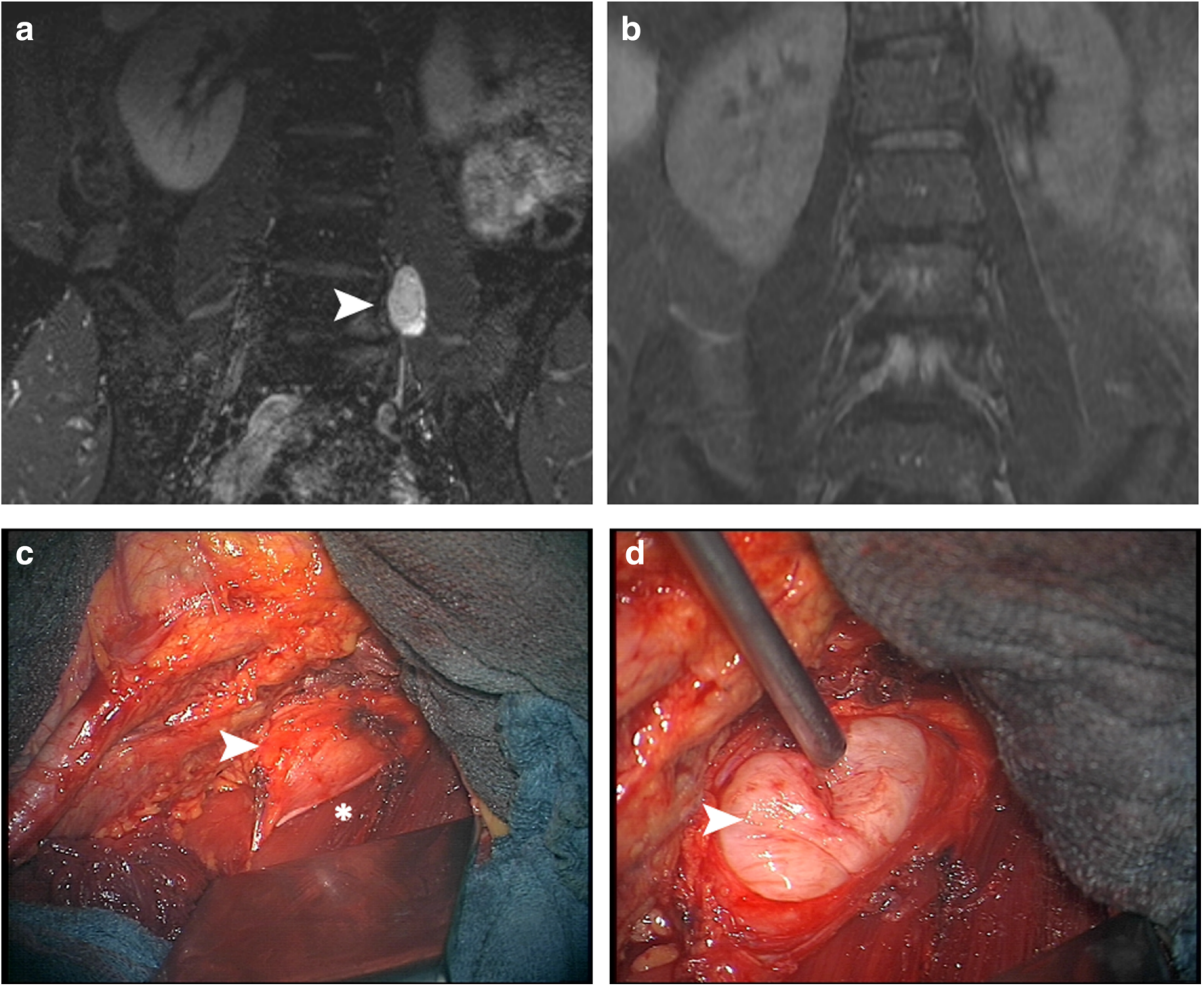
Pre- (**A**) and postoperative (**B**) MRI of a retroperitoneal PNST (white arrow). Once the general surgeon had exposed the tumor behind the psoas muscle (*), the neurosurgeon incised the pseudocapsule (white arrow in **C**), dissected the plane between the pseudocapsule and the true tumor capsule and removed the tumor (white arrow in **D**)

The general surgeon actively participated during the entire surgery and first, chose a suitable approach to the tumor, performed the laparotomy, and identified/mobilized vital structures such as the ureter, the intestine, and the large iliac blood vessels (Fig. 1). As soon as the tumor was exposed by the general surgeon, the neurosurgeon incised the pseudocapsule, dissected the plane between the pseudocapsule and the true tumor capsule, and removed the tumor. In the event of bleeding from a ruptured iliac vessel or in the case of organ perforation, the general surgeon would take over temporarily. After tumor resection, the general surgeon sewed up the laparotomy.

### Preoperative patient data

We assessed pain and neurological function prior to surgical treatment, and recorded any forgoing treatment or fine-needle biopsy. In addition, we gathered T1 and T2 weighted MRI imaging sets to measure preoperative tumor size and assessed tumor localization.

### Intraoperative patient data

Intraoperative data included the surgical approach, potential intraoperative surgical complications, extent of tumor resection, operative time, intraoperative blood loss, and where applicable, intraoperative blood transfusion.

### Postoperative patient data

Postoperative follow-up included tumor histology, duration of hospital stay, postoperative surgical complications according to the Clavien-Dindo classification [7], changes of preoperative symptoms, postoperative neurological function**,** and tumor burden on MRI at clinical follow-up in our outpatient clinic.

### Data analysis

All statistical analyses were done using GraphPad Prism version 8.0.0 for Mac, GraphPad Software, La Jolla California USA, www.graphpad.com. D’Agostino-Pearson K2 normality test was performed to assess normal distribution of our data. Where applicable and depending on normal distribution of data, a paired *t* test or a Wilcoxon matched-pairs signed rank test was performed. For correlation of data sets, and depending on normal distribution of data, Pearson or Spearman’s correlation coefficient was calculated. A *p* value of less than 0.05 indicated a statistically significant difference.

## Results

### Preoperative patient characteristics

The patient characteristics are summarized in Table 1. In total, *n* = 9/18 patients presented with unilateral pain radiating into the lower extremity and *n* = 2/18 patients complained of lower back pain. For the remaining patients (*n* = 7/18), tumor diagnosis was incidental.

**Table 1:** Extended patient characteristics

Gender	Age (year)	Histology	Preoperative needle biopsy?	Tumor status at follow-up	Preoperative symptoms	Symptoms after surgery	Follow-up (days)
f	62	Schwannoma WHO I°	No	NA	Pain radiating into lower extremity	No improvement	Lost to follow-up
M	30	Schwannoma WHO I°	Yes	NA	None	NA	Lost to follow-up
M	53	Schwannoma WHO I°	No	Tumor-free	Pain radiating into lower extremity	Pain relief	96
M	61	Schwannoma WHO I°	No	Tumor-free	Pain radiating into lower extremity	Pain relief	244
M	64	Schwannoma WHO I°	No	NA	None	NA	3358
M	62	Schwannoma WHO I°	No	Recurrence	None	NA	369
F	35	Schwannoma WHO I°	No	Tumor-free	None	NA	761
M	41	Schwannoma WHO I°	No	Tumor-free	Lower back pain	No improvement	356
F	67	Schwannoma WHO I°	Yes	Tumor-free	None	Transient numbness of lower extremity	455
F	20	Schwannoma WHO I°	No	NA	None	NA	Lost to follow-up
F	28	Ganglioneuroma WHO I°	No	Static tumor remnant	None	NA	85
M	49	Neurofibroma WHO I°	No	Tumor-free	Pain radiating into lower extremity	Pain relief, sub-ileus symptoms	82
F	52	Neurofibroma WHO I°	No	Tumor-free	Pain radiating into lower extremity	Pain relief	110
F	23	Neurofibroma WHO I°	Yes	NA	Pain radiating into lower extremity	Pain relief	Lost to follow-up
M	53	Schwannoma WHO I°	Yes	Tumor-free	Pain radiating into lower extremity	Pain relief	110
F	31	Schwannoma WHO I°	No	Tumor-free	Lower back pain	Pain relief	3500
F	23	Malignant peripheral nerve sheath tumor	No	Progressive tumor	Pain radiating into lower extremity	No improvement	630
M	55	Malignant peripheral nerve sheath tumor	No	Progressive tumor	Pain radiating into lower extremity	Pain relief	511

*NA*, not applicable

Mean tumor volume was 65.9 cm^3^ (95% CI 24.1–107.7 cm^3^). In total, *n* = 15/18 tumors were located paravertebrally and presacrally and the remaining *n* = 3/18 tumors were growing into the foramen obturatum.

Preoperative CT-guided needle biopsy was not routinely employed, except in four cases, where retroperitoneal tumors could not be classified on preoperative MRI scans (Table 1). Biopsy histology identified PNSTs in 4/4 patients (3 schwannomas and 1 neurofibroma) and was later confirmed by intraoperative histology in all cases.

### Surgical treatment and intraoperative data

Total tumor removal was achieved in *n* = 14/16 PNST patients. Subtotally resected tumors comprised one ganglioneuroma, which could not be safely removed from the tumor-bearing nerve and one schwannoma with inaccessible intraforaminal tumor burden at the L5 nerve root.

An additional patient presented with suspected retroperitoneal PNST on preoperative MRI scans and was ultimately diagnosed with MPNST. This patient was not diagnosed with core-needle biopsy and was subjected to microscopic, nerve-sparing tumor resection. Postoperative MRI showed minimal, residual contrast enhancement. Due to the small tumor burden, the local tumor board recommended radiation treatment.

Another patient suffered from lymphoma and had previously received systemic treatment. After presenting with a retroperitoneal tumor, and given the likelihood of lymphoma recurrence, he had been offered CT-guided biopsy, but opted for open biopsy and partial tumor debulking instead.

Mean operative time for our interdisciplinary approach was 162.5 min (95% CI, 121.6–203.4 min). There were no major surgical or anesthesiologic complications during surgery. In *n* = 3/18 cases, tumors were located in direct contact with iliac vessels and in two of these cases, a ruptured branch of the inner iliac vein was sewed by the general surgeon. Assistance of a vascular surgeon was not required in these cases. In the fourth case, the bladder was discretely torn during tumor removal and required sewing by the general surgeon. After tumor resection, the general surgeon took over to sew up the laparotomy. In one patient, due to the suspected risk of incisional hernia, the general surgeon performed a hernioplasty. Mean blood loss was 680.6 ml (95% CI, 194.3–1167.0 ml).

### Postoperative data and follow-up

Pathologies included schwannomas (*n* = 12), neurofibromas (*n* = 3), ganglioneuroma (*n* = 1) and MPNST (*n* = 2). Necrosis was only seen in MPNST (*n* = 2/2) and in one case of schwannoma with increased tumor cell proliferation as inferred by frequency of Ki-67-positive cells. While mean proliferation rates in the remaining schwannomas was 6.0% (95% CI, 2.7–9.3%), this particular tumor was diagnosed with 20% of proliferating tumor cells, a Ki-67 rate comparable to the mean proliferation rate of MPNST (22.5%). There were, however, no other particularities associated with this case of schwannoma and the corresponding patient remained tumor-free at follow-up (Table 1). Of note, there was no correlation between tumor cell proliferation and tumor size at diagnosis (Spearman correlation coefficient = 0.20; *p* = 0.43).

Mean hospital stay was 9.8 days (95% CI, 8.6–10.9 days). There were no major postoperative surgical complications. One patient suffered from transient sub-ileus symptoms but required no further intervention (grade I complication according to Clavien-Dindo Classification [7]).

A total of 8/11 patients with preoperative pain reported durable improvement of their pain symptoms (Table 1). The remaining patients reported no improvement. One patient suffered from a transient postoperative numbness of the lower extremity, which regressed shortly after surgery and did not restrict the patient in her daily life.

We contacted patients and, if necessary, primary physicians, to schedule follow-ups at our outpatient clinic. In total, *n* = 4/18 patients (22%) were lost to follow-up, specifically, two patients moved out of the country and could not be reached and one patient died from causes unrelated to the tumor. One patient could not be reached despite repeated efforts. Altogether, *n* = 14/18 patients were amenable to follow-up. After a mean follow-up of 761.9 days (95% CI, 97.6–1426.0 days), all but two PNST patients were tumor-free (Table 1). Both of the PNST patients, who had been subjected to partial tumor resection, were diagnosed with a static tumor remnant at follow-up.

One of the two MPNST patients presented 15 months after initial surgery with retroperitoneal and intra-/paraspinal-tumor progression. She was subjected to dorso-ventral tumor resection, again, by an interdisciplinary team. Specifically, the intraspinal tumor parts at L4-S1 were removed by the neurosurgeon via hemilaminectomy, including removal of the tumor-infiltrated S1 nerve root. Following resection of these tumor parts, the general surgeon performed a radical removal of the paraspinal and retroperitoneal tumor parts via laparotomy. Despite these efforts, the patient suffered tumor progression 3 months later and, in the later course of the disease, died due to renal failure.

In the second patient, following MPNST diagnosis, the local tumor board recommended radiotherapy followed by systemic therapy with doxycycline due to the patient’s general condition.

## Discussion

PNST are benign tumors that rarely recur after tumor removal and have no impact on a patient’s life expectancy or quality of life. As such, guidelines generally recommend nerve-sparing PNST removal. Retroperitoneal PNST, however, are rare, difficult to diagnose preoperatively, and challenging to operate [3, 8]. In addition, retroperitoneal PNST are treated by surgeons from a variety of disciplines such as visceral surgery, oncological surgery, urology, and gynecology, which reduces the average number of cases that any given surgeon will see in a lifetime to just a handful of tumors.

Given the rareness of retroperitoneal PNST, their proximity to vital visceral organs and blood vessels as well as potential differential diagnoses such as sarcomas, there is no standard treatment for retroperitoneal PNST.

As such, it is difficult to conceive a prospective study comparing radical and nerve-sparing retroperitoneal PNST resection in terms of neurological outcome, surgical complications, and oncological outcome. Therefore, our study and previous reports are limited to retrospective case series and any comparison between studies should bear this limitation in mind.

Initially, we hypothesized that our interdisciplinary approach was best suited to improve preoperative symptoms and maintain neurological function. So far, previous reports have neglected to systematically study neurological outcome, which is crucial given that retroperitoneal PNST originate from lumbosacral nerve roots or the lumbar plexus and thus might require careful, nerve-sparing tumor resection, especially in cases where patients present with neurological symptoms. In our series, retroperitoneal tumor resection was associated with a low, transient neurological morbidity of approximately 6% (*n* = 1/18 patients) and more than two-thirds of patients reported lasting pain relief after surgery. Even though we are not aware of other studies systematically reporting neurological outcome after retroperitoneal PNST removal, we think that this favorable neurological outcome speaks in favor of performing a microscopic, nerve-sparing resection.

We further considered, whether our minimally invasive tumor resection was associated with less surgical morbidity, e.g., less intraoperative blood loss, which has been consistently identified as a major intraoperative problem in previous studies [5, 9].

During our interdisciplinary approach, dissection of major blood vessels was kept at a minimum, especially since PNST often adhere to major blood vessels. Once the tumor pseudocapsule had been identified, the neurosurgeon continued the operation, incised the pseudocapsule, and extirpated the tumor, thus reducing the risk of intraoperative hemorrhage. It is important to mention that in *n* = 3/18 cases, tumors were in direct contact with iliac vessels and required careful mobilization of vessels to expose the tumor. Once the tumor pseudocapsule was incised, tumors could be removed in safe distance from the iliac vessels, i.e., the pseudocapsule served as plane separating the tumor site from blood vessels. Still, in two of these cases, ruptured branches of the inner iliac vein, which were draining the tumor, had to be sewed by the general surgeon. In one of these cases, major blood loss (3800 ml) led to blood transfusion. Assistance of vascular surgeons, however, was not required. To our knowledge, other studies have not systematically studied blood loss after retroperitoneal PNST removal, but incidents with massive, sudden blood loss [5, 9] and tearing of adjacent arteries have been reported 8]. In our series, mean intraoperative blood loss was 680.6 ml (95% CI, 194.3–1167.0 ml) and was comparable to blood loss reported after open resection of other benign retroperitoneal tumors [10].

We did not record any intraoperative surgical or anesthesiologic complications and our low postoperative surgical morbidity was in line with complication rates reported in other studies on retroperitoneal PNST resection [8, 9, 11] and in studies on resection of other benign retroperitoneal tumor entities [10].

Additionally, mean operative time for our interdisciplinary approach was 162.5 min (95% CI, 121.6–203.4 min). While other studies have not systematically studied surgery durations, our mean operative times are comparable to that of other surgical studies reporting on resection of benign retroperitoneal tumor entities. Liu et al., for instance, reported mean operative times of 152.7 min ± SD 72.4 min for open resection of benign retroperitoneal tumors, including PNST [10]. We thus hypothesize that our interdisciplinary approach is, at least, comparable to “classical” retroperitoneal PNST resection in terms of surgical morbidity and that our interdisciplinary approach is not more time-consuming than traditional, monodisciplinary surgery.

At the same time, gross total tumor removal could be achieved in 88% of our PNST patients (*n* = 14/16 patients), which is well in line with two other studies, which reported complete tumor removal rates of 73% (*n* = 60/82 patients) [9] and 85% (*n* = 17/20 patients) [11]. In the former study, tumor enucleation was followed by tumor (pseudo) capsule removal. In the latter study, the surgical method was not explicitly mentioned. Most likely though, “classical” tumor resection was performed, as one patient underwent resection of a contiguous organ and one patient received only subtotal resection due to tumor adherence to the iliac vein. Finally, a third study reported a complete resection rate of 100% after en bloc tumor resection but included 7 patients only [8].

In terms of follow-up after complete PNST removal, all totally resected patients were tumor-free at follow-up and patients that had been subjected to partial tumor resection were diagnosed with static tumor remnants. With a mean follow-up of 24.9 months, our study is within the follow-up range of other clinical series, and oncological results are in line with other studies on retroperitoneal PNST. Reported rates of recurrence after total resection have been as low as 1.2% (*n* = 1/81 patients, mean follow-up 63 months) [9] and 0% (0/7 patients, mean follow-up 17 months) [8]. In the former study, stable tumor burden after partial tumor resection was reported for 8/8 patients after a mean follow-up of 9.5 years.

Finally, and in contrast to another report [8], preoperative CT-guided needle biopsy in our study was accurate in 4/4 cases, i.e., biopsy results were confirmed by intraoperative pathology. We will thus continue to employ CT-guided needle biopsies in cases with unclear retroperitoneal pathology. In addition, we will expand their use in the light of new and/or updated guidelines for the treatment of sarcoma [6] and especially, in order to avoid cases of unplanned MPNST resections.

We must point out, though, that biopsy recommendations in current sarcoma guidelines are primarily based on studies focusing on tumors of the trunk and of the extremities [12–14]. Some of these studies did not include retroperitoneal tumors or excluded patients with impalpable, deep-seated lesions from their final analysis [13], and others included a small number of retroperitoneal tumors only [12]. At the same time, Dupuy et al. reported that biopsy accuracy depended on tumor localization and reported a mismatch rate of 20% in the spinal and paraspinal region [15]. In these cases, biopsies had retrieved unrepresentative tissue and radiologists arguably felt less comfortable using longer and larger needles and performing multiple passes [15].

Altogether, we believe that there is little knowledge on the diagnostic accuracy of core-needle biopsies in cases of retroperitoneal tumors, and guideline recommendations are based primarily on experiences with sarcomas of the trunk and of the extremities.

Finally, a main advantage of our interdisciplinary approach for PNST resection is the possibility to move from a microsurgical, nerve-sparing resection to a more aggressive, oncological resection in those rare cases were MPNST can be diagnosed with intraoperative histology. And even in cases with unplanned MPNST resection, re-resection seems to be feasible without influencing overall survival [16].

## Conclusion

Interdisciplinary, nerve-sparing enucleation of retroperitoneal PNST is well suited to improve preoperative symptoms and maintain neurological function, while achieving results similar to other series on radical retroperitoneal PNST resection, in terms of oncological outcome, surgical morbidity, and operative times.
